# Reviewers BJCVS 32.5

**Published:** 2017

The Brazilian Journal of Cardiovascular Surgery (BJCVS) is grateful for the reviewers, listed below, which collaborated in this edition. Without their work would be impossible to keep the high scientific standard of our journal.

Alexandre Hueb
Douglas Bolzan
Eduardo K. Saadi
Fernando Platania
Gabriel Liguori
Gibran Feguri
João Carlos Ferreira Leal
João de Deus e Brito
José Carlos M. Pachon
Jose Dario Frota Filho
Karlos Vilarinho
Lindemberg Silveira
Luciano Albuquerque
Luís Dallan
Magaly Arrais dos Santos
Marcos Aurelio Barboza de Oliveira
Michel Pompeu B. de O. Sá
Milton Santoro
Paulo Roberto Evora
Pedro Salerno
Renato Arnoni
Ricardo Carvalho Lima
Rodrigo Milani
Stevan Martins

